# Biomechanical Assessment During High‐Resolution Impedance Manometry Enhances Characterization of Inconclusive Esophagogastric Junction Outflow Obstruction

**DOI:** 10.1111/nmo.70450

**Published:** 2026-10-07

**Authors:** Aidan D. Smires, Wenjun Kou, John E. Pandolfino, Panyavee Pitisuttithum, Eric Goudie, Muhammed M. Alikhan, Neelesh A. Patankar, Dustin A. Carlson

**Affiliations:** ^1^ Kenneth C. Griffin Esophageal Center of Northwestern Medicine, Department of Medicine, Division of Gastroenterology and Hepatology, Feinberg School of Medicine Northwestern University Chicago Illinois USA; ^2^ Center of Excellence in Neurogastroenterology and Motility, Division of Gastroenterology, Department of Medicine, Faculty of Medicine Chulalongkorn University Bangkok Thailand; ^3^ Division of Thoracic Surgery, Department of Surgery Université de Montréal Montreal Quebec Canada; ^4^ Department of Mechanical Engineering, McCormick School of Engineering Northwestern University Evanston Illinois USA

**Keywords:** dysphagia, electric impedance, esophageal achalasia, esophageal motility disorders, esophagogastric junction, manometry

## Abstract

**Background:**

Esophagogastric junction outflow obstruction (EGJOO) identified on high‐resolution manometry (HRM) remains a clinically heterogeneous and inconclusive finding. Additional complementary testing with functional lumen imaging probe (FLIP) or timed barium esophagram (TBE) is recommended to confirm clinically relevant obstruction. We aimed to assess the association of novel Four‐Dimensional (4D) HRM metrics against FLIP and TBE outcomes among symptomatic patients with inconclusive EGJOO on HRM.

**Methods:**

Adult patients with inconclusive HRM‐EGJOO who completed corresponding FLIP and TBE studies were identified for inclusion in a cross‐sectional, retrospective study. Primary 4D HRM metrics of EGJ opening, EGJ distensibility index (4D EGJ‐DI), and 4D maximum EGJ diameter were analyzed relative to reference outcomes defined by FLIP and esophagram.

**Results:**

Among 123 patients (mean age 59 years; 60% female), 4D EGJ‐DI and 4D maximum EGJ diameter differed significantly across FLIP and TBE outcome categories (all *p* < 0.01). Lower 4D EGJ‐DI and lower 4D maximum EGJ diameter were associated with reduced EGJ opening on FLIP and abnormal retention on esophagram. There were 0 patients with normal EGJ opening on FLIP that had 4D maximum EGJ diameter < 8.0 mm and 0 patients with reduced EGJ opening on FLIP that had a 4D EGJ‐DI > 2.2 mm^2^/mmHg and 4D maximum EGJ diameter > 10.2 mm.

**Conclusions:**

By integrating biomechanical assessment of EGJ opening with conventional pressure metrics, 4D HRM provides complementary physiologic information for evaluating esophageal motor function. This additional information may improve characterization of patients with inconclusive EGJOO.

## Key Points

1. Esophagogastric junction outflow obstruction (EGJOO) identified on high‐resolution manometry (HRM) per Chicago Classification v4.0 is a non‐specific, inconclusive finding that requires complementary testing to confirm clinically relevant obstruction.
2. Four‐dimensional (4D) HRM derives biomechanical metrics of EGJ opening, distensibility, and maximum diameter from the same standard HRM study, offering complementary physiologic information without an additional procedure.
3. 4D EGJ metrics showed consistent relationships with FLIP and TBE outcomes, supporting the potential use of 4D HRM to add biomechanical information to standard HRM and help contextualize inconclusive EGJOO.

## Graphical abstract

In 123 patients with inconclusive esophagogastric junction outflow obstruction, 4D EGJ‐DI and diameter differed significantly across FLIP and TBE groups and reliably discriminated normal from reduced EGJ opening on FLIP.

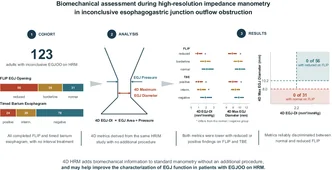

## 1 Introduction

A high‐resolution manometry (HRM) classification of esophagogastric junction outflow obstruction (EGJOO) represents a common and often challenging clinical finding in gastroenterology [1, 2, 3, 4]. The Chicago Classification version 4.0 (CCv4.0) defines the HRM pattern of EGJOO when there is elevated integrated relaxation pressure (IRP) and peristalsis (i.e., peristalsis present such that a conclusive achalasia pattern is not observed) with increased intrabolus pressure (IBP), and associated obstructive symptoms (e.g., dysphagia, chest pain) [1, 2]. The clinical challenge arises as this HRM pattern is non‐specific and can be observed across heterogeneous clinical scenarios that include a variant‐form of achalasia, opioid use, mechanical obstructions (e.g., hiatal hernia or subtle stricture), or even a false positive HRM related to pressure artifact [1, 2].

Because of the clinical uncertainty associated with the EGJOO finding isolated to HRM, CCv4.0 recommended utilization of additional testing with barium esophagram or functional lumen imaging probe (FLIP) to confirm (or refute) the clinical relevance of the HRM findings, especially prior to pursuing invasive, achalasia‐type treatments [1, 2, 5]. While FLIP directly assesses EGJ opening mechanics, that is, distensibility and diameter during controlled volume‐distension, TBE measures a *consequence* of EGJ obstruction (retention), but does not directly measure EGJ function. Hence, these tests should not be viewed as equivalent measures of EGJ obstruction, though they frequently are. Further, both esophagram and FLIP carry their own inherent limitations, including patient logistic challenges and delayed diagnosis (additional patient visit to complete). Further, while these tests may support “obstruction,” they do not perfectly determine the underlying mechanism for obstruction, for example, impaired LES relaxation (achalasia) vs. mechanical obstruction. Overall, seeking approaches to better address the conundrum of EGJOO on HRM remains an area of ongoing investigation.

Four‐Dimensional High‐Resolution Impedance Manometry (4D HRM) is a novel technique that utilizes impedance to provide an estimation of luminal cross‐sectional area (CSA) during the standard Chicago Classification HRM protocol when performed with a high‐resolution impedance manometry (HRIM) assembly [6, 7]. The term 4D was not intended to be taken literally, but was chosen to reflect the utilization of a fourth domain of luminal CSA provided by the 4D HRM approach time, in addition to the three standard domains from HRM (time; space/axial length; and pressure). Thus, while conventional HRM characterizes pressure generation but provides only indirect information regarding luminal opening during swallowing, 4D HRM provides a novel approach to integrate biomechanics by calculating esophageal lumen diameter and distensibility (i.e., CSA divided by pressure) during esophageal bolus transit as it occurs during the HRM test protocol.

Previous studies demonstrated that 4D HRM metrics of EGJ distensibility were abnormal in achalasia and correlated with FLIP metrics across a spectrum of conclusive esophageal motility diagnoses (i.e., achalasia and normal motility), as well as correlating with achalasia treatment outcomes including timed barium esophagram (TBE) [7, 8, 9]. Thus, we hypothesized that 4D HRM would add biomechanical information to standard HRM that helps contextualize inconclusive EGJOO. This study aimed to assess how 4D HRM metrics of EGJ distensibility and opening were associated with FLIP and TBE outcomes among a cohort of symptomatic patients with inconclusive EGJOO on HRM.

## 2 Methods

### 2.1 Subjects

Adult patients (aged 18–89) presenting to the Esophageal Center of Northwestern for evaluation of esophageal symptoms between December 2014 and June 2024 had data prospectively maintained in an esophageal motility registry. After excluding for previous foregut surgery (including POEM and pneumatic dilation) and overt mechanical obstruction (e.g., hiatal hernia > 3 cm, Los Angeles C‐D erosive esophagitis, stricture, eosinophilic esophagitis), the data registry was queried for patients who completed corresponding (i.e., no interval treatment between tests) HRM, FLIP, and TBE and had an HRM classification of EGJOO per CCv4.0 [1, 2]. HRMs completed prior to 2020 had been reviewed to reflect the updated Chicago Classification v4.0 criteria for EGJOO. Technical limitations of HRIM studies were also cause for exclusion; Figure S1. There is overlap of this cohort with previous publications [5, 10].

The study protocol was approved by the Northwestern University Institutional Review Board as minimal risk with a waiver of informed consent for analysis of deidentified, coded patient data.

### 2.2 HRIM Protocol

A 4.2‐mm outer diameter solid‐state HRIM assembly with 36 circumferential pressure sensors at 1‐cm interval, and 18 impedance segments at 2‐cm intervals (Medtronic Inc., Shoreview, MN) was utilized. HRIM studies were performed after at least 6 h of fasting. The assembly was placed transnasally and positioned to record from the hypopharynx to the stomach with about three intragastric pressure sensors. The HRIM study was performed and interpreted per Chicago Classification version 4.0 [1]. The protocol included ten 5 mL liquid supine swallows and five 5 mL upright swallows using 50% saline. A median IRP of > 15 mmHg was considered abnormal for supine swallows; a median IRP of > 12 mmHg was considered abnormal for upright swallows. Elevated IBP was determined when there was compartmentalized pressure > 20 mmHg (using the isobaric contour tool) between the peristaltic wave and the EGJ.

### 2.3 4D HRM Analysis

4D HRM analysis was performed blinded to clinical characteristics including FLIP and TBE findings. For each HRIM study, the first 5 supine swallows with adequate impedance readings were analyzed to derive 4D HRM metrics. As previously described in detail, data from each swallow in Manoview software were exported into a customized analysis software [6, 7, 8]. Landmarks were manually input to delineate the four phases of esophageal bolus transport: accommodation (Phase 1), compartmentalization (Phase 2), peristalsis/esophageal emptying (Phase 3), and ampullary emptying (Phase 4); Figure 1 [8, 11]. Phase‐specific calculations of intraluminal CSA were derived from impedance data based on bolus properties, Ohm's law, and mass conservation, with CSA transformed to diameter (to facilitate interpretation) with assumption of a circular lumen. Analysis focused on measures of 4D maximum EGJ diameter and pressure at the maximum EGJ diameter during the peristalsis/esophageal emptying phase (i.e., Phase 3) as bolus emptying across the EGJ primarily occurs during this phase. The 4D HRM EGJ‐DI from Phase 3 was then calculated as the maximum EGJ CSA divided by pressure, similar to the approach used in FLIP analysis. While initial landmark placement requires manometry competence, subsequent 4D metric calculation follows an automated algorithmic pipeline, facilitating use by trained fellows and physicians. Upright swallows were not analyzed given their strong correlation with supine swallow findings. Exclusion of inadequate swallows or studies was primarily due to impedance sensor malfunction. Inter‐rater reliability and further methodological detail have been previously reported [8].

**FIGURE 1:**
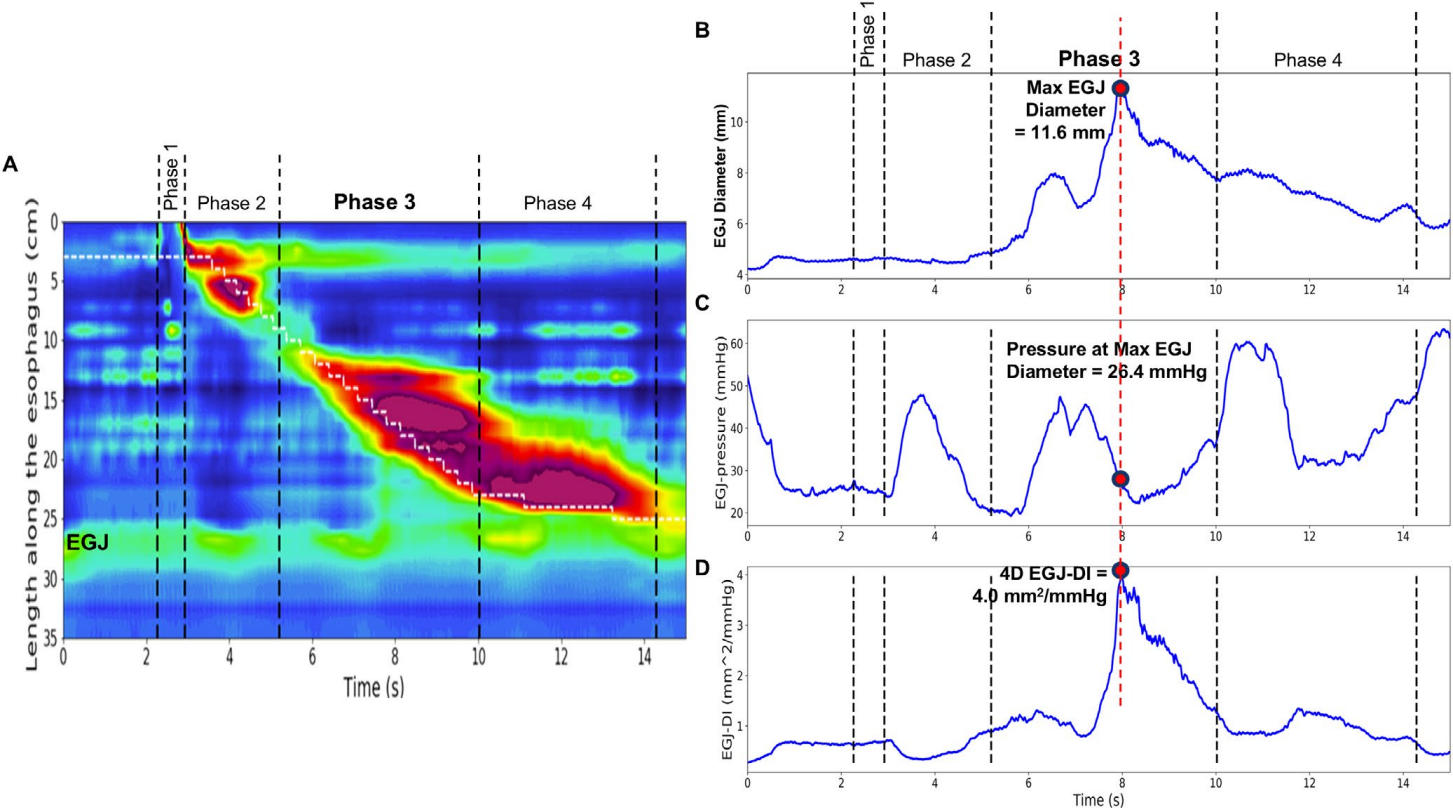
Four dimensional (4D) high‐resolution manometry (HRM). A representative swallow from a patient with EGJ outflow obstruction on HRM is displayed. This patient had normal EGJ opening on FLIP and normal barium clearance on timed barium esophagram. The four phases of bolus transit are illustrated by vertical dashed lines in panels A–D. The time history of (B) 4D Esophagogastric Junction (EGJ) Diameter. (C) EGJ pressure (C), and (D) EGJ distensibility index (EGJ‐DI) are also displayed. The 4D EGJ‐DI is calculated at the time point of the max EGJ diameter during phase 3 by integrating the associated EGJ pressure, as represented by the red‐dashed vertical line and red circle in each panel (B–D). Figure used with permission from the Esophageal Center of Northwestern.

### 2.4 FLIP Protocol and Analysis

The FLIP study was performed during sedated endoscopy using a 16‐cm FLIP catheter (EndoFlip EF‐322N; Medtronic) and analyzed as previously described [10]. Briefly, the FLIP was placed transorally and positioned in the esophagus with 1–3 impedance sensors beyond the EGJ. FLIP positioning was confirmed at the 30–40 mL fill volume and then 10 mL stepwise FLIP distension was performed to 50 mL, 60 mL, and 70 mL with each stepwise distension volume maintained for 30–60 s. FLIP data were exported using a customized program (available open source at https://www.wklytics.com/nmgi) to generate FLIP Panometry plots for analysis. Analysis of the EGJ focused on the EGJ‐distensibility index (DI) at the 60 mL FLIP fill volume and the maximum EGJ diameter achieved during the 60 or 70 mL fill volume. EGJ opening was then classified as reduced EGJ opening (REO: EGJ‐DI < 2.0 mm^2^/mmHg and maximum EGJ diameter < 12 mm; positive FLIP result), borderline EGJ opening (BEO: not reduced nor normal EGJ opening; intermediate FLIP result), or normal EGJ opening (NEO: EGJ‐DI ≥ 2.0 mm^2^/mmHg and maximum EGJ diameter ≥ 16 mm; negative FLIP result) [12, 13].

### 2.5 TBE Protocol and Analysis

Timed barium esophagram (TBE) was performed by having the patient drink 200 mL of thin barium while in an upright position with anteroposterior images obtained at 1, 2 and 5 min after ingestion. The barium column height above the EGJ was measured at each time point. If liquid barium cleared, a 12.5 mm barium tablet was also administered. TBE results of liquid emptying were categorized for analysis based on the findings of greatest severity as 5‐min column height > 5 cm (positive TBE); 1‐min column height > 8 cm (intermediate TBE); or “normal” (negative TBE) if the 1‐min column was ≤ 8 cm. Additional analysis was performed for whether the 12.5 mm barium tablet passed or not (i.e., impacted) among studies in which the tablet was used. TBE thresholds were chosen based on previously defined thresholds where likelihood ratios are increased to detect achalasia or conclusive EGJOO [14].

### 2.6 Statistical Analysis

Results were reported as mean (standard deviation [SD]) or median (interquartile range [IQR]) depending on the data distribution. Correlations were assessed using Spearman's rho. Differences between more than two independent groups were compared using the Kruskal–Wallis test. When a significant overall difference was identified, pairwise comparisons were performed using the Mann–Whitney *U* test. A two‐sided *p*‐value < 0.05 was considered statistically significant. Post hoc comparison testing was completed using a Bonferroni correction.

While the Chicago Classification recommends utilization of FLIP *or* TBE in the setting of inconclusive EGJOO on HRM, FLIP was utilized as the primary outcome as both the 4D metrics and FLIP metrics directly assess EGJ function, thus providing a more like‐to‐like comparison. TBE was applied as a secondary outcome, particularly among cases with 4D HRM and FLIP discordance, noting that retention on TBE is associated with EGJ outflow obstruction, but is an indirect measure as peristaltic function and esophageal anatomy also affect esophageal clearance. To evaluate the diagnostic utility of 4D HRM metrics across clinically meaningful measurement intervals, interval likelihood ratios (ILRs) were calculated for the FLIP EGJ opening classification. Recognizing the clinical uncertainty associated with BEO on FLIP, separate analyses were performed with all BEO classified as negative (i.e., grouped with NEO on FLIP), and then separately with BEO on FLIP classified as positive (i.e., REO on FLIP). ILRs build upon standard positive and negative likelihood ratios by partitioning the range of continuous HRM metrics into discrete intervals, preserving more information than a binary threshold while remaining clinically interpretable. For each 4D HRM metric, the continuous range was divided into pre‐specified intervals based on clinical thresholds. Adjacent intervals were then merged as needed to ensure a monotonic relationship in ILRs across the distribution. Within each interval, the ILR was computed as the ratio of the proportion of outcome‐positive patients falling within that interval to the proportion of outcome‐negative patients falling within the same interval. ILRs > 1 indicate that a measurement falling within that interval is associated with an increased post‐test probability of a positive outcome, ILRs less than 1 indicate that the interval is associated with a reduced post‐test probability, and an ILR of 1 implies no change in probability. In contrast to the prespecified ILR intervals, the combined 4D EGJ‐DI and 4D maximum EGJ diameter thresholds used in the multimetric analysis were derived from exploratory inspection of the study cohort and were not prespecified.

## 3 Results

### 3.1 Subjects

123 patients, mean (SD) age 59 (15) years, 60% female with HRM‐EGJOO (inconclusive) were included; Table 1; Figure S1. The primary indication for evaluation was dysphagia in 89%, chest pain in 5%, or reflux‐like symptoms in 6%, though all had reported dysphagia or chest pain. Endoscopy findings among the cohort included small hiatal hernia in 28 patients (23%) and Los Angeles grade A esophagitis in 2 patients. The median (IQR) IRP in the supine position was 23 (19–32) mmHg and in the upright position was 23 (18–31) mmHg. 20 patients (16%) were on chronic opioids.

**TABLE 1:** Cohort characteristics.

Patient characteristics	
*n* (%)	123 (100)
Age, mean (SD) years	59 (15)
Sex; *n* (%) female	74 (60)
Primary Indication for evaluation, *n* (%)	
Dysphagia	110 (89)
Chest pain	6 (5)
Reflux symptoms	7 (6)
HRM findings	
HRM, median supine IRP, mmHg, median (IQR)	23 (19–32)
HRM, median upright IRP, mmHg, median (IQR)	23 (18–31)
HRM, Basal EGJ pressure (end‐expiration), mmHg, median (IQR)	28 (18–37)
HRM EGJ morphology, *n* (%)	
Type I	71 (58)
Type II	49 (40)
Type III	3 (2)
HRM‐primary peristalsis/contractility pattern, *n* (%)	
Normal	55 (45)
Ineffective	35 (29)
Hypercontractile	9 (7)
Premature	24 (20)
FLIP results	
EGJ‐distensibility index (DI), mm^2^/mmHg, median (IQR)	1.6 (0.8–2.8)
Maximum EGJ diameter, mm, median (IQR)	12.4 (9.6–16.4)
Timed Barium Esophagram (TBE) results	
1 min column height, cm, median (IQR)	3.9 (0–12)
5 min column height, cm, median (IQR)	0 (0–0.9)
Tablet impaction, *n* (% with tablet used)	24 (27)

Abbreviations: FLIP, functional lumen imaging probe; HRM, high‐resolution manometry; IRP, integrated relaxation pressure; TBE, timed barium esophagram.

Time between HRIM and FLIP was median (IQR) 0 (0–25) days and between HRIM and TBE was median (IQR) 34 (8–70) days. FLIP was positive (REO) in 56 patients (46%), intermediate (BEO) in 36 (29%), and negative (NEO) in 31 (25%). TBE was positive (abnormal) in 24 patients (20%), intermediate in 29 (24%), and negative (normal) in 70 (57%). A barium tablet was used in 88 cases: tablet impaction occurred in 24 (27%; 17/24, 70% also had liquid retention) and tablet passage occurred in 64 (73%; 10/64, 16% had liquid retention).

### 3.2 FLIP Outcomes

The 4D EGJ‐DI was significantly correlated with FLIP EGJ‐DI (rho = 0.510) and FLIP maximum EGJ diameter (rho = 0.481); *p*‐values < 0.001; Figure S2. The 4D maximum EGJ diameter was also significantly correlated with FLIP EGJ‐DI (rho = 0.435) and FLIP maximum EGJ diameter (rho = 0.424); *p*‐values < 0.001.

Additionally, 4D EGJ‐DI (*p* < 0.001) and 4D maximum EGJ diameter (*p* < 0.001) differed across FLIP EGJ opening classes; Figure 2. Both 4D EGJ‐DI and 4D maximum EGJ diameter were significantly lower in REO than in BEO or NEO on FLIP. There was not a significant difference in either 4D EGJ‐DI or 4D maximum EGJ diameter between BEO and NEO (4D EGJ‐DI: adj. *p* = 1.0; 4D maximum EGJ diameter: adj. *p* = 0.55).

**FIGURE 2:**
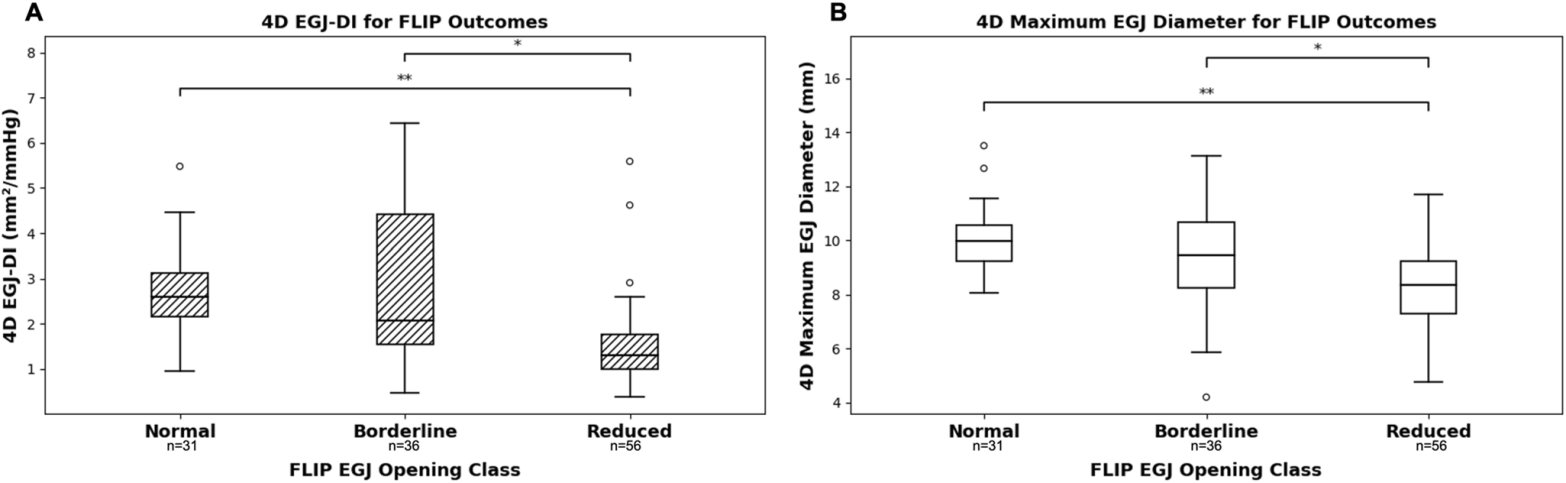
4D HRM metrics by FLIP outcomes among inconclusive EGJOO on HRM. Box plots display (A) 4D EGJ distensibility index (EGJ‐DI) and (B) 4D maximum EGJ diameter across FLIP EGJ opening classifications: Open circles denote outliers. Brackets indicate significant pairwise comparisons after Bonferroni correction (*adj. *p* < 0.05; **adj. *p* < 0.001). 4D HRM, four‐dimensional high‐resolution manometry; EGJOO, esophagogastric junction outflow obstruction; FLIP, functional lumen imaging probe.

An increase in likelihood (i.e., ILR > 1.0) for having abnormal EGJ opening on FLIP was observed for 4D EGJ‐DI < 1.0 mm^2^/mmHg (ILR 5.4–5.6) and for 4D EGJ‐DI 1.0–2.0 mm^2^/mmHg (ILR 1.8–2.6), whereas a 4D EGJ‐DI > 2.0 mm^2^/mmHg indicated a reduced likelihood of REO on FLIP (ILR 0.3–0.4), Table 2. An increase in likelihood (i.e., ILR > 1.0) for having abnormal EGJ opening on FLIP was also observed for 4D maximum EGJ diameter < 8.0 mm (ILR 3.1), with zero patients with NEO on FLIP having 4D maximum EGJ diameter < 8.0 mm (resulting in ‘undefined’ ILR). 4D maximum EGJ diameter 8.0–10 mm (ILR 0.7–0.9) and 4D maximum EGJ diameter > 10 mm (ILR 0.4–0.6) indicated a reduced likelihood of abnormal EGJ opening on FLIP; Table 2.

**TABLE 2:** Interval Likelihood Ratios (ILR) for 4D HRM Metrics.

	Outcome (intermediate as “negative”)			Outcome (intermediate as “positive”)		
4D HRM metric and intervals						
4D EGJ‐DI (mm^2^/mmHg)	FLIP+, n	FLIP‐neg, n	ILR	FLIP+, n	FLIP‐neg, n	ILR
< 1.0	14	3	5.6	16	1	5.4
1.0–2.0	32	31	1.8	47	6	2.6
> 2.0	10	43	0.3	29	24	0.4
4D Maximum EGJ Diameter (mm)	FLIP+, n	FLIP‐neg, n	ILR	FLIP+, n	FLIP‐neg, n	ILR
< 8.0	23	9	3.1	32	0	undef.
8–10	23	30	0.9	36	17	0.7
> 10	10	28	0.4	24	14	0.6

Abbreviations: FLIP, functional lumen imaging probe; HRM, high‐resolution manometry.

### 3.3 TBE Outcomes

4D EGJ‐DI (*p* < 0.001) and 4D maximum EGJ diameter (*p* = 0.002) differed by TBE categories; Figure 3. 4D EGJ‐DI was lower in positive TBE and intermediate TBE than negative TBE. 4D maximum EGJ diameter was also lower in positive TBE than negative TBE. 4D EGJ‐DI and 4D maximum EGJ diameter were both lower in patients with tablet impaction than in those with tablet passage; Figure 3.

**FIGURE 3:**
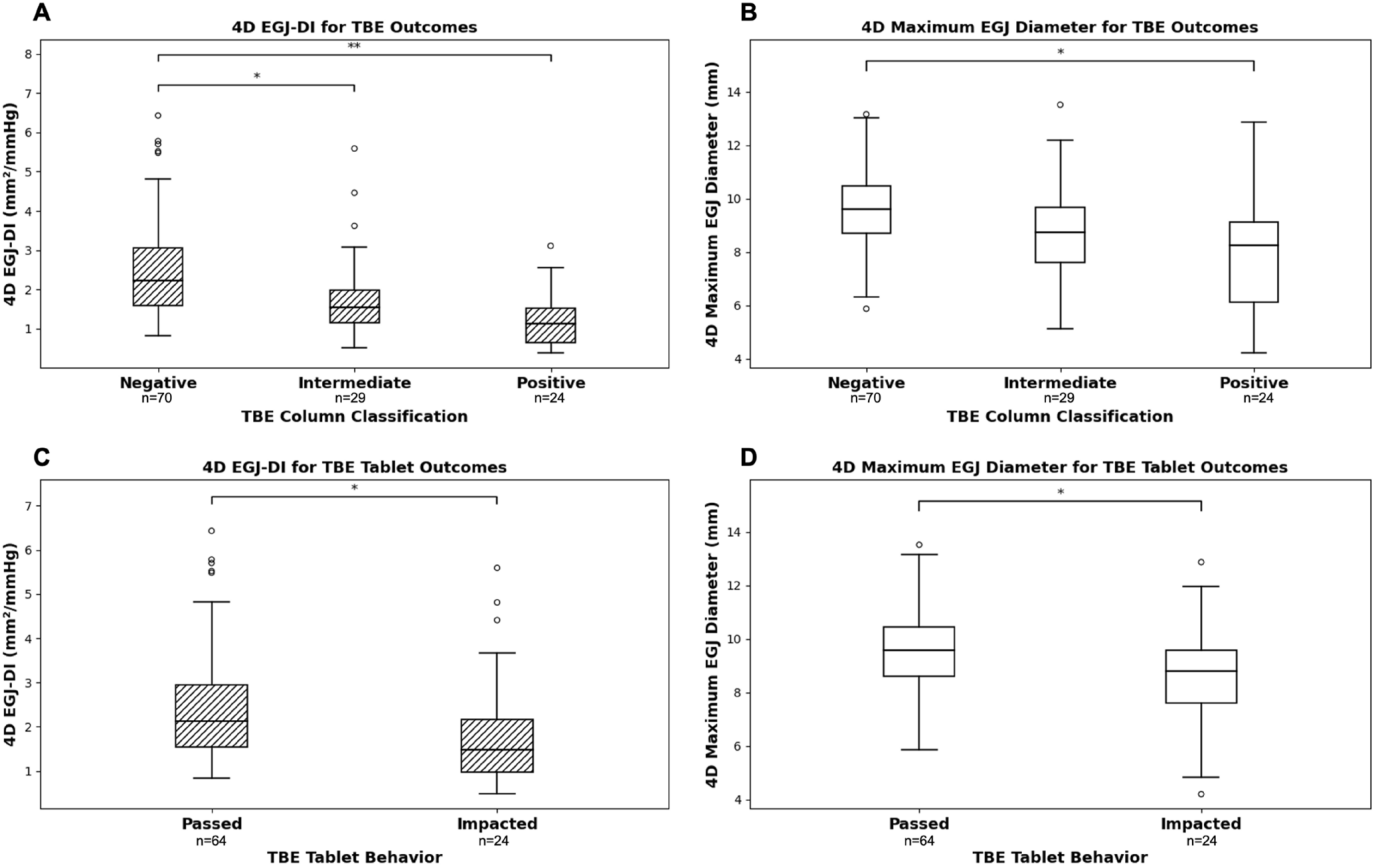
4D HRM metrics by TBE outcomes and barium tablet passage among inconclusive EGJOO on HRM. Box plots display 4D EGJ distensibility index (EGJ‐DI) and 4D maximum EGJ diameter stratified by timed barium esophagram (TBE) findings. (A, B) Metrics by TBE liquid column classification. (C, D) Metrics by barium tablet behavior among studies in which a tablet was used. Open circles denote outliers. Brackets indicate significant pairwise comparisons after Bonferroni correction (*adj. *p* < 0.05; **adj. *p* < 0.001). 4D HRM—four‐dimensional high‐resolution manometry; EGJOO, esophagogastric junction outflow obstruction; TBE, timed barium esophagram.

### 3.4 Multimetric Analysis

Finally, the relationships of 4D EGJ‐DI and 4D maximum EGJ diameter were assessed relative to FLIP outcomes, using TBE outcomes secondarily in discordant cases: Figure 4. There were 0 patients with NEO on FLIP that had a 4D maximum EGJ diameter < 8.0 mm: Figure 4A. Additionally, there were 0 patients with REO on FLIP that had both a 4D EGJ‐DI > 2.2 mm^2^/mmHg and a 4D maximum EGJ diameter > 10.2 mm: Figure 4C. Among BEO on FLIP (inconclusive FLIP and HRM), there were 10 patients with 4D EGJ‐DI > 2.2 mm^2^/mmHg and a 4D maximum EGJ diameter > 10.2 mm, zero of whom had significant liquid retention on TBE (with 90% having normal TBE; 10% intermediate TBE) and only 2 (22%) that had tablet impaction: Figure 4B. Also, among BEO on FLIP, there were nine patients with 4D maximum EGJ diameter < 8 mm: 1 (11%) had a positive TBE and 3 (33%) had tablet impaction: Figure 4B.

**FIGURE 4:**
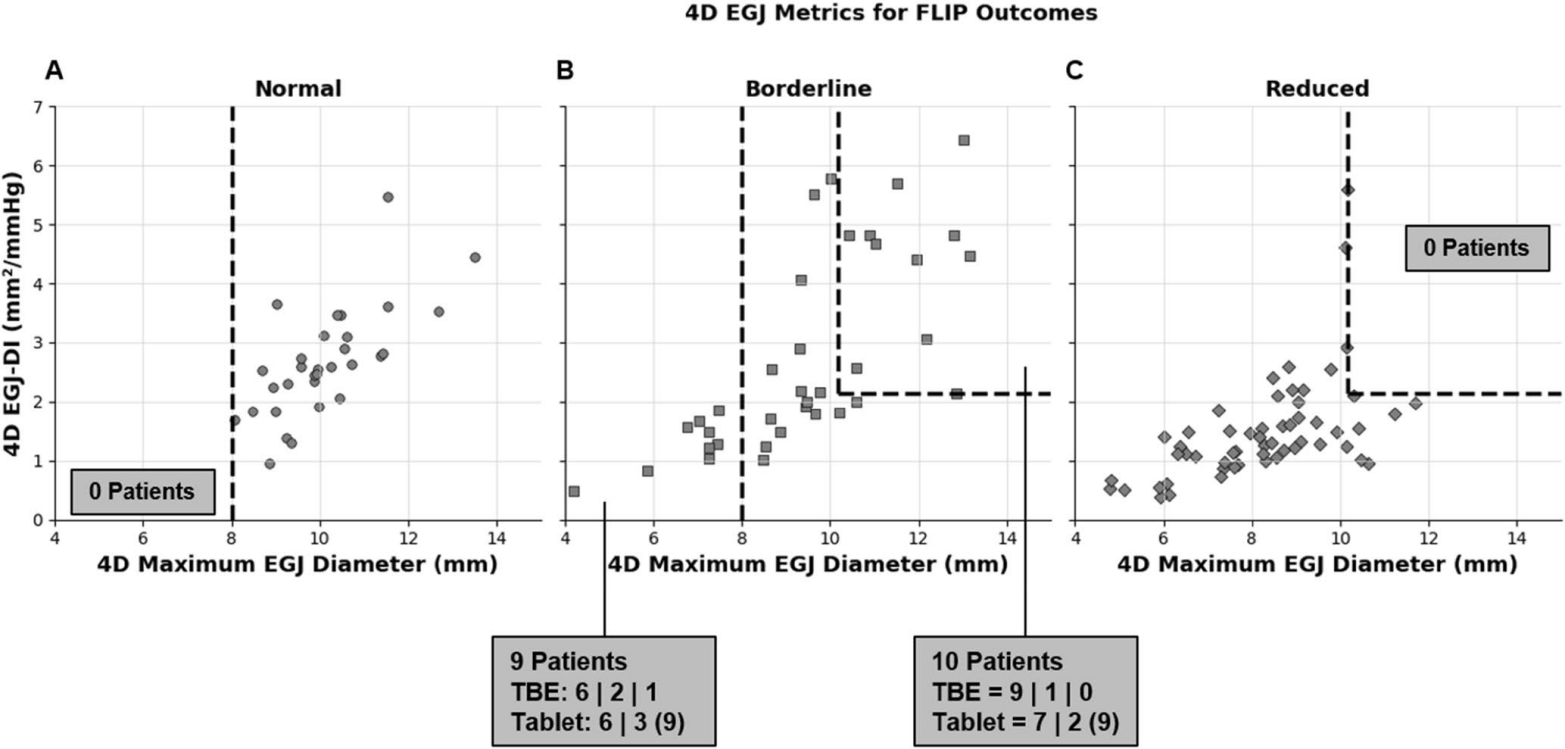
Relationship of combined 4D HRM EGJ metrics with FLIP EGJ opening classifications among patients with inconclusive EGJOO on HRM. Each data point represents a single patient with panels designated by FLIP EGJ opening category. (A) Normal EGJ opening. (B) Borderline (inconclusive) EGJ opening. (C) Reduced EGJ opening. TBE outcome formatted as Negative | Intermediate | Positive. Tablet outcome formatted as Passed | Impacted (Total patients with tablet used). 4D HRM—four‐dimensional high‐resolution manometry; EGJOO—esophagogastric junction outflow obstruction; FLIP—functional lumen imaging probe.

## 4 Discussion

This study demonstrates that 4D HRM metrics obtained during standard CCv4.0 HRIM test protocol can help stratify the likelihood of clinically relevant EGJOO among patients with ‘inconclusive’ (based on HRM alone) EGJOO. Specifically, 4D HRM metrics of 4D EGJ‐DI and 4D maximum EGJ diameter showed consistent relationships with FLIP and TBE outcomes, with clinically interpretable probability intervals (ILRs; Table 2). Further, when 4D HRM showed 4D EGJ‐DI > 2.2 mm^2^/mmHg and 4D maximum EGJ diameter > 10.2 mm (Figure 4), FLIP and/or TBE findings rarely supported relevant EGJOO. These findings support that 4D HRM provides clinically relevant biomechanical information beyond conventional pressure‐based HRM metrics and may help contextualize inconclusive EGJOO findings (e.g., elevated IRP) identified during standard CCv4.0 evaluation.

EGJOO remains one of the most clinically challenging classifications within the CCv4.0 framework as this HRM pattern is clinically non‐specific and can arise from a variety of etiologies [2]. Consequently, CCv4.0 recommends additional, complementary testing with FLIP or TBE prior to pursuing invasive, achalasia‐type interventions. Notably, these tests measure different aspects of esophageal function—namely, FLIP can measure EGJ opening in response to volume‐controlled distension while TBE or barium tablet observed with fluoroscopy assesses esophageal retention (i.e., an indirect surrogate for “obstruction”)–yet either is recommended to confirm (or refute) relevant EGJ obstruction. Unlike conventional HRM, which primarily characterizes pressure patterns, 4D HRM quantifies luminal opening and bolus transport mechanics during physiologic swallowing using the same HRIM study already obtained during routine evaluation. Thus, 4D HRM offers the potential to augment diagnostic confidence without requiring an additional procedure.

The observed stepwise decrease in 4D EGJ‐DI and 4D maximum EGJ diameter across FLIP‐defined EGJ opening categories, among this cohort that universally had elevated IRP values from standard HRM (by design), reinforces the physiologic validity of these metrics. This parallel between 4D HRM and FLIP distensibility thresholds may be expected as both metrics assess EGJ biomechanics using lumen CSA normalized to intraluminal pressure. However, as reflected by the modest correlations, this is not an apples‐to‐apples comparison as 4D HRM derives CSA from impedance during bolus transit during a swallow (e.g., 5 mL liquid test swallow) whereas the FLIP study assesses the EGJ response to applied volumetric distension. Hence while LES relaxation is involved during both, and both assess biomechanical EGJ opening, there are inherent physiologic and mechanical differences to the processes being measured.

That said, when assessing the clinical relevance of FLIP findings, “NEO” on FLIP carries a very high negative predictive value for relevant EGJ outflow obstruction and thus offers significant potential to “rule out” achalasia‐type pathology [12, 13]. Key findings in this study were that when 4D HRM had a maximum EGJ diameter < 8 mm, there were no patients with NEO on FLIP, thus a scenario in which relevant EGJOO is not ruled out, increased likelihood for conclusive EGJOO remains, and additional testing may carry higher yield. However, when 4D maximum EGJ diameter was > 10.2 mm and 4D EGJ‐DI was > 2.2 mm^2^/mmHg, that is, both 4D metrics utilized together, REO on FLIP was not observed (and a minority with BEO on FLIP had abnormal TBE findings). Hence, this appeared to support a scenario where relevant EGJOO may reliably be refuted. On the other hand, REO on FLIP is associated with achalasia; however, an entity of “distension mediated obstruction” in which FLIP shows REO and HRM shows normal LES relaxation also exists [13, 15, 16]. While “distension mediated obstruction” remains a novel finding of clinical interest, its underlying mechanisms and best clinical approaches still remain uncertain. Thus, parallel findings of reduced EGJ distensibility and opening on both 4D HRM and FLIP may lend support to the relevance of EGJ outflow obstruction, though likely remains a scenario in which additional complementary testing with TBE and tablet precedes pursuit of aggressive LES‐disruptive treatments such as myotomy.

Further, borderline EGJ opening on FLIP (i.e., a clinically inconclusive finding), in the setting of an inconclusive HRM, poses additional clinical uncertainty. In this scenario, this study suggests that integrating 4D HRM findings may help add clarity, particularly with normal‐range 4D EGJ‐DI and 4D maximum EGJ diameter. Among the subgroup in this study with BEO on FLIP and a 4D EGJ‐DI > 2.2 mm^2^/mmHg and a 4D maximum EGJ diameter > 10.2 mm (values suggestive of preserved distensibility), the majority demonstrated normal clearance on TBE bolus transit, with only 1 showing “intermediate” liquid retention on TBE and 2 (22%) experiencing tablet impaction (Figure 4B). This suggests that reassuring 4D metrics may help identify patients whose borderline FLIP result reflects true functional adequacy, supporting a more conservative management approach. It is also important to acknowledge that the relationship between abnormal 4D metrics (4D maximum EGJ diameter < 8 mm) and abnormal bolus transit in this borderline group was less consistent, thus likely identifying a set of findings that warrants additional assessment with TBE with tablet and/or a cautious therapeutic approach.

This study has multiple strengths, including the use of objective reference standards with both FLIP and TBE, a focus on a clinically challenging population, and the application of a quantitative and clinically interpretable framework. However, there are also limitations to consider. First, the reference standards (FLIP and TBE) are imperfect, and each contains intermediate and potentially discordant categories. This was addressed by utilizing FLIP and TBE categories that focused on the more distinctly normal or abnormal findings for each outcome. However, it also underscores that discordance between these various tests should be expected as they measure different aspects of esophageal function, and their ultimate application should be interpreted in a complementary manner within a global clinical context to guide management decisions. An additional limitation is that this was a single‐center study performed at a tertiary esophageal referral center, which may limit generalizability to broader practice settings. Other potential contributing factors, such as opioid use, hiatal hernia, and HRM contractility pattern, were captured but not adjusted for in sensitivity or multivariable analyses, given the risk of overfitting in a single‐center cohort of this size. Further, clinical outcomes and treatment response data were not assessed, so the ability of 4D HRM metrics to predict therapeutic response and to directly guide clinical decisions remains unclear. We are in the process of developing open‐source software for 4D HRM analysis that will facilitate future broader, multicenter studies to further test, validate, and refine the techniques and thresholds described here.

In conclusion, 4D HRM provides novel, physiologically relevant metrics that may improve the characterization of EGJ function in patients with inconclusive EGJOO on standard HRM by incorporating biomechanical evaluation of EGJ opening. While 4D HRM did not appear to represent a replacement for FLIP or TBE, which provide complementary physiologic information under different testing conditions, 4D HRM provided additional physiologic information that can aid clinical interpretation and may help identify patients most likely to benefit from complementary testing. Additionally, 4D HRM metrics may help understand discordant or borderline FLIP and TBE findings. Ultimately, 4D HRM appears to be a promising advance in the evaluation of esophageal motility disorders, and further validation is warranted in larger prospective cohorts with treatment outcome assessment.

## Author Contributions

Aidan D. Smires contributed to drafting of the manuscript, data analysis, data interpretation, and approval of the final version. Wenjun Kou contributed to data analysis, data interpretation, editing the manuscript critically, and approval of the final version. John E. Pandolfino contributed to study concept and design, obtaining funding, editing the manuscript critically, data interpretation, and approval of the final version. Panyavee Pitisuttithum, Eric Goudie, Muhammed M. Alikhan, and Neelesh A. Patankar contributed to data analysis and approval of the final version. Dustin A. Carlson contributed to study concept and design, obtaining funding, drafting of the manuscript, data analysis, data interpretation, and approval of the final version.

## Funding

This work was supported by R01 DK137775 (John E. Pandolfino and Dustin A Carlson) from the Public Health service (NIDDK).

## Conflicts of Interest

John E. Pandolfino: Medtronic (Speaking, Consulting, Patent, License); Sandhill Scientific/Diversatek (Consulting, Speaking, Grant); Torax (Speaking, Consulting); EndoGastric Solutions (speaking; consulting); Laborie (Consulting). Wenjun Kou: Calyx, Sanofi (Consulting). Dustin A. Carlson: Medtronic (Speaking, Consulting, License); Diversatek (Consulting); Braintree (Consulting); Medpace (Consulting); Phathom Pharmaceuticals (Speaking; Consulting); Regeneron/Sanofi (Speaking, Consulting); Laborie (Consulting).

## Supporting information


**Figure S1:** Inclusion/Exclusion flow diagram for cohort of EGJ outflow obstruction (EGJOO) on high‐resolution manometry (HRM) per Chicago Classification version 4.0 (CCv4.0).
**Figure S2:** 4D and FLIP EGJ metric correlation scatter plots. Each point represents a single patient, colored by FLIP EGJ opening class: normal (open circles), borderline (gray squares), and reduced (black triangles). Panels plot 4D HRM metrics against FLIP metrics for each pairing: (A) 4D EGJ‐DI versus FLIP EGJ‐DI, (B) 4D EGJ‐DI versus FLIP maximum EGJ diameter, (C) 4D maximum EGJ diameter versus FLIP EGJ‐DI, and (D) 4D maximum EGJ diameter versus FLIP maximum EGJ diameter. FLIP, functional lumen imaging probe.

## Data Availability

The data that support the findings of this study are available on request from the corresponding author.
