# Supplementary material for: Biomechanical Assessment During High‐Resolution Impedance Manometry Enhances Characterization of Inconclusive Esophagogastric Junction Outflow Obstruction
Source: Neurogastroenterol Motil. 2026 Oct 7;38(10):e70450. doi: 10.1111/nmo.70450 (PMC13646348; doi:10.1111/nmo.70450)
Supplement: Supplementary file 1 [file NMO-38-e70450-s001.docx]

**Supplementary Material**

**Figure S1. Inclusion/Exclusion flow diagram for cohort of EGJ outflow obstruction (EGJOO) on high-resolution manometry (HRM) per Chicago Classification version 4.0 (CCv4.0).**

**Figure S2. 4D and FLIP EGJ metric correlation scatter plots.** Each point represents a single patient, colored by FLIP EGJ opening class: normal (open circles), borderline (gray squares), and reduced (black triangles). Panels plot 4D HRM metrics against FLIP metrics for each pairing: (A) 4D EGJ-DI versus FLIP EGJ-DI, (B) 4D EGJ-DI versus FLIP maximum EGJ diameter, (C) 4D maximum EGJ diameter versus FLIP EGJ-DI, and (D) 4D maximum EGJ diameter versus FLIP maximum EGJ diameter. FLIP – functional lumen imaging probe.
